# Supplementary material for: All-inorganic perovskite quantum dot light-emitting memories
Source: Nat Commun. 2021 Jul 22;12:4460. doi: 10.1038/s41467-021-24762-w (PMC8298456; doi:10.1038/s41467-021-24762-w)
Supplement: Supplementary file 1 — Supplementary Information [file 41467_2021_24762_MOESM1_ESM.pdf]

Supporting Information

All-inorganic perovskite quantum dot light-emitting memories

Meng-Cheng Yen, Chia-Jung Lee, Kang-Hsiang Liu, Yi Peng, Junfu Leng, Tzu-Hsuan Chang, Chun-Chieh Chang, Kaoru Tamada, and Ya-Ju Lee

M. -C. Yen, C. -J. Lee, K.-H. Liu, Y. Peng, Prof. C.-C. Chang, Prof. Y.-J. Lee
Institute of Electro-Optical Engineering, National Taiwan Normal University, 88, Sec. 4, Ting-Chou Rd., Taipei, 11677, Taiwan
E-mail: chang48@ntnu.edu.tw ; yajulee@ntnu.edu.tw

Dr. J. Leng, Prof. K. Tamada
Institute for Materials Chemistry and Engineering (IMCE), Kyushu University, Fukuoka 819-0395, Japan

Prof. K. Tamada
Advanced Institute for Materials Research (AIMR), Tohoku University, Sendai 980-8577, Japan
E-mail: tamada@ms.ifoc.kyushu-u.ac.jp

Prof. T.-H. Chang
Graduate Institute of Electronics Engineering, National Taiwan University, No. 1, Sec. 4, Roosevelt Rd., Taipei, 10617, Taiwan

Supplementary Figure 1 | $CsPbBr_3$ bulk-based RRAM device

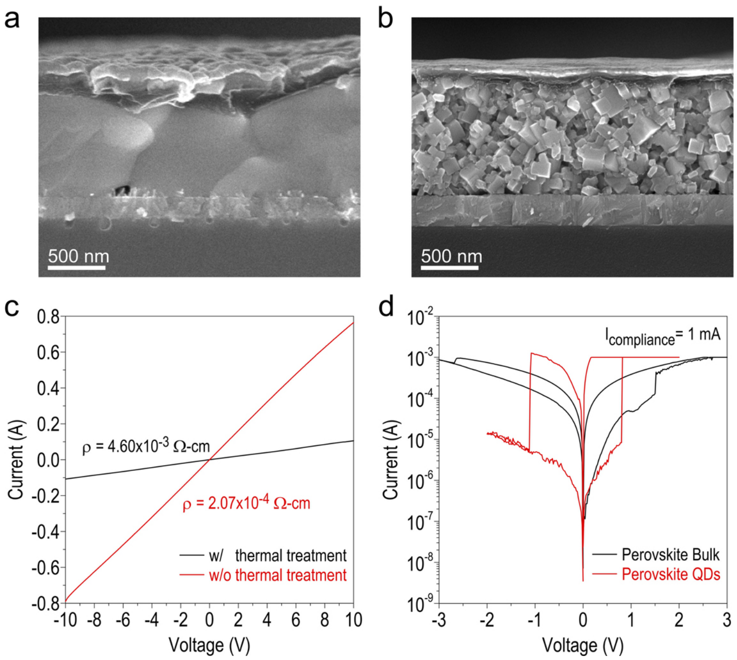

Supplementary Figure 1 Cross-sectional SEM image of the RRAM device based on a **a** bulk and a **b** quantum-dot perovskite active layer, respectively. **c** Measured resistivity of the patterned ITO electrodes with (black line) and without (red line) the thermal treatment at 650 °C for 10 mins. **d** I-V characteristics of the RRAM with a bulk (black curve) and a quantum-dot (red curve) active layer.

Supplementary Note 1

Supplementary Figure 1a shows the cross-sectional SEM image of the $CsPbBr_3$ RRAM employing a bulk perovskite active layer grown by a vapor phase approach in a furnace system. The glass substrate (area = 1.5 cm × 1.5 cm) with patterned ITO electrodes is placed downstream of a quartz tube located in the furnace center. Two alumina boats loaded respectively with $PbBr_2$ and CsBr powders as precursors are put in the heating center of the tube. Prior to heating, the quartz tube is pumped down to 0.02 Torr by a mechanical pump, and then purged with high-purity nitrogen at a flow rate of 50 sccm to maintain a pressure of 600 Torr. The furnace is then heated to 650 °C in ~ 30 mins and stayed there for 10 mins. After that, the furnace is cooled down naturally to room temperature before removing the sample. As shown in the SEM, the grown $CsPbBr_3$ bulk

is ~ 1 μm thick. The PMMA layer and the Ag electrode are subsequently deposited on top of the $CsPbBr_3$ bulk layer to complete the RRAM fabrication. For comparison, a $CsPbBr_3$ QD-based RRAM is also prepared at room temperature (the QD layer thickness is also ~ 1μm), with its cross-sectional SEM image shown in Supplementary Figure 1b. As the measured resistivity of the patterned ITO electrodes increases from $\rho = 2.07 \times 10^{-4}$ to $4.60 \times 10^{-3}\ \Omega \cdot \mathrm{cm}$ after the thermal treatment (Supplementary Figure 1c), both the set (1.52 V) and reset (– 2.64 V) voltages for the RRAM device using the $CsPbBr_3$ bulk active layer are higher than those for the QD-based device (set: 0.82 V, reset: – 1.12 V, Supplementary Figure 1d). The ON/OFF current ratio of the $CsPbBr_3$ bulk-based RRAM is also reduced to be ≤ 10. Therefore, the $CsPbBr_3$ QDs are preferred over the bulk in this work as the active layer, since the entire process for synthesizing the $CsPbBr_3$ QDs is conducted at room temperature, and thus the possible influence of thermal stress on the RRAM performance, such as the increased resistivity of the underlying patterned ITO electrodes clearly shown here, can be eliminated.

Supplementary Figure 2 | Quantum confinement effect of $CsPbBr_3$ QDs

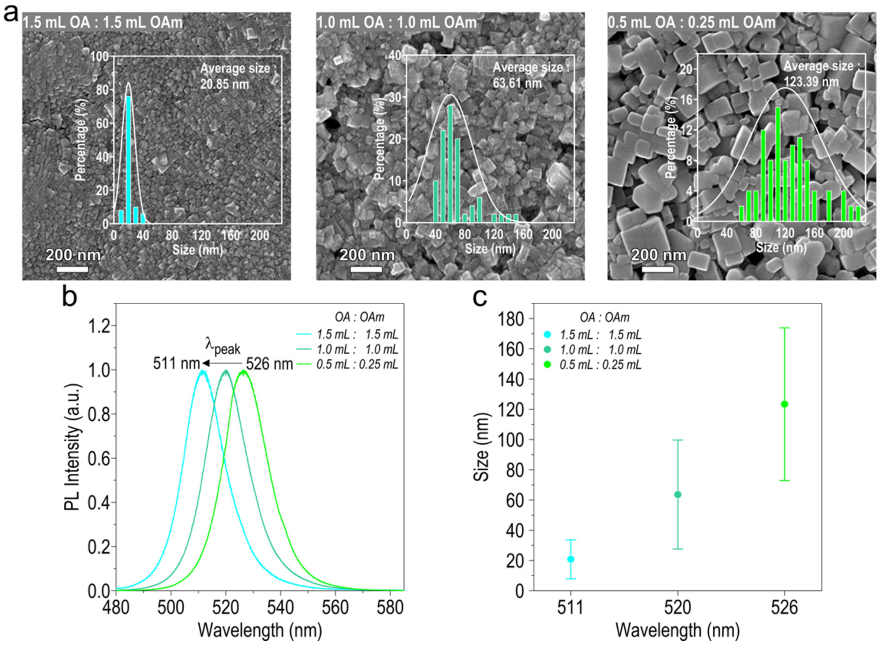

Supplementary Figure 2 a Top-view SEM images of the $CsPbBr_3$ QDs with different sizes obtained by varying the OA: OAm ratios of the stabilization solution during the synthesis. The size distributions of the synthesized dots (in percentage) are also inserted in the images. The scale bars are 200 nm in all SEMs. **b** PL spectra of the synthesized $CsPbBr_3$ QDs with different sizes. **c** Size dependence of the $CsPbBr_3$ QDs on the peak emission wavelength of their PL spectra. Error bars represent plus/minus standard deviation.

Supplementary Note 2

To explore the tunable bandgaps of the $CsPbBr_3$ resulting from the quantum confinement effect and their importance in the $CsPbBr_3$ QD-based LEM functionalities, we systematically reduce the size of the $CsPbBr_3$ QDs by changing the mixture ratios between the Oleic acid (OA) and the oleylamine (OAm) during the stabilization process for the $PbBr_2$/CsBr precursor solution. The average sizes of the synthesized $CsPbBr_3$ QDs (all with the same stoichiometry) are 123, 63, and 20 nm, for the OA: OAm ratios of 0.5 ml: 0.25 ml, 1.0 ml: 1.0 ml, and 1.5 ml:1.5 ml, respectively (Supplementary Figure 2a). The size distributions of the synthesized $CsPbBr_3$ QDs (in percentage) are also estimated and inserted in the figures. Clearly, gradually reducing the QD size blueshifts the peak emission wavelength of the PL spectra, from 526 to 520, and then 511 nm, due to the increased

quantum confinement effect (Supplementary Figure 2b and 2c). The demonstrated tunable bandgaps of the $CsPbBr_3$ QDs offer a great opportunity to significantly enhance the LEM device functionalities, as evidenced by the realization of the two-color emitting LEM device employing two different QDs (see Figure 7 in the main text).

Supplementary Figure 3 | Top-view SEM images of $CsPbBr_3$ QD-based LEM

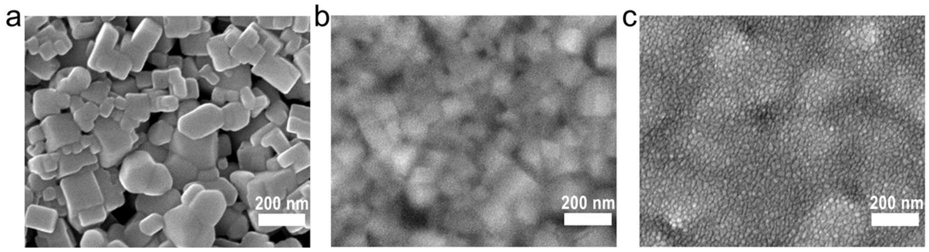

Supplementary Figure 3 Top-view SEM images of **a** $CsPbBr_3$ QDs, **b** PMMA/$CsPbBr_3$ QDs, and **c** Ag/PMMA/$CsPbBr_3$ QDs. The scale bars are 200 nm in all images.

Supplementary Note 3

Supplementary Figure 3 shows top-view SEM images of the $CsPbBr_3$ QDs (left panel), the PMMA/$CsPbBr_3$ QDs (middle panel), and the Ag/PMMA/$CsPbBr_3$ QDs (right panel). The PMMA protection layer in the device structure provides an effective way to prevent the Ag penetration during the sputtering deposition of Ag, which in turn minimizes the formation of current leakage paths between the top (Ag) and the bottom (ITO) electrodes, and thus enhances the performance and the stability of the fabricated LEM.

Supplementary Figure 4 | AFM images of $CsPbBr_3$ QD-based LEM

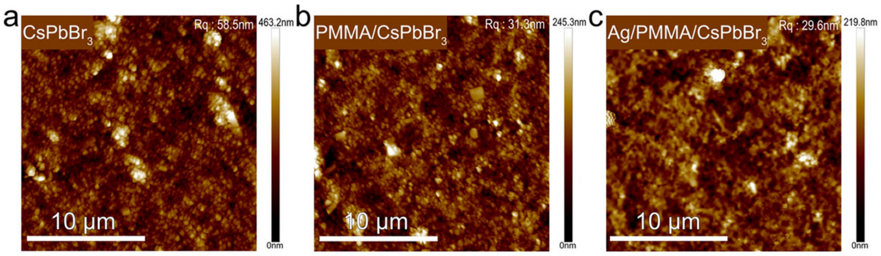

Supplementary Figure 4 AFM images of **a** $CsPbBr_3$ QDs, **b** PMMA /$CsPbBr_3$ QDs, and **c** Ag/PMMA/$CsPbBr_3$ QDs films. The measured root-mean-square surface roughness (R_q) values are shown in the upper right corner of each image. The scale bars are 10 μm in all images.

Supplementary Note 4

Supplementary Figure 4 shows AFM images of the $CsPbBr_3$ QDs, the PMMA /$CsPbBr_3$ QDs, and the Ag/PMMA/$CsPbBr_3$ QDs films. Their corresponding root-mean-square surface roughness values are measured to be 58.5, 31.3, and 29.6 nm, respectively. Clearly, the PMMA planarizes the surface of the $CsPbBr_3$ QD layer, very likely by filling the voids between the aggregated dots. After the subsequent deposition of a 100-nm-thick Ag film on top, the measured surface roughness is almost identical to that of the PMMA /$CsPbBr_3$ QDs films, further validating the importance of using the PMMA protection layer for enhancing the performance and stability of the fabricated LEM device.

Supplementary Figure 5 | Migration tendency of Ag and Br in $CsPbBr_3$ QD-based RRAM

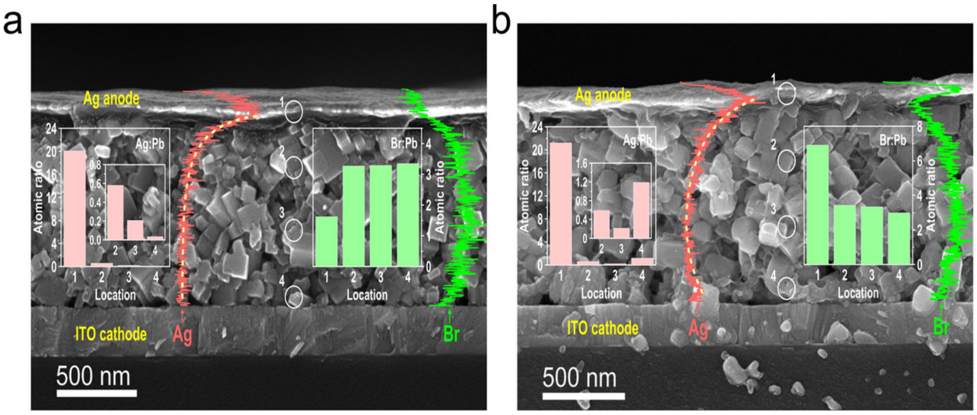

Supplementary Figure 5 Migration tendency of Ag and Br observed in the cross-sectional SEM images of the Ag/PMMA/$CsPbBr_3$ QDs/ITO stack **a** without and **b** with a positive poling bias of 2 V. Cross-sectional distributions of Ag (red curve) and Br (green curve) from top to bottom are also plotted in both figures. Insets: column graphs of the Ag: Pb and Br: Pb ratios measured at different locations denoted as 1, 2, 3, and 4.

Supplementary Note 5

We investigate the ion migrations responsible for the observed I-V characteristics in the LEM by using the SEM measurement and EDS analysis. Supplementary Figure 5 shows the cross-sectional SEM images of the Ag/PMMA/$CsPbBr_3$ QDs/ITO stack a without and b with a positive poling bias of 2 V (Ag: anode, ITO: cathode). Cross-sectional distributions of Ag (red curve) and Br (green curve) are plotted in both figures to elucidate the migration tendency of the dissociated cations and anions inside the $CsPbBr_3$ QD layer. For both samples, four different locations denoted as 1 – 4 from the top to the bottom electrode are selected to qualitatively inspect the changes of the atomic ratios (including Ag: Pb and Br: Pb) due to the poling bias, and the results are also plotted as column graphs inserted in the figures. For the sample without the poling bias (Supplementary Figure 5a), the highest density of Ag is found at the location 1 near the top electrode (Ag: Pb ratio ~ 20.08), and the detected Ag signal decreases gradually along the detecting depth, corresponding to a monotonous decline of the Ag: Pb ratio from ~ 0.58 (location 2), ~ 0.21 (location 3) to ~ 0.03 (location 4). After applying the positive poling bias on the RRAM (Supplementary

Figure 5b), the detected Ag signal basically follows a similar trend, except that in the location 4 close to the device bottom, a high Ag density with an enhanced Ag: Pb ratio of ~ 1.20 is detected. It validates that the Ag^+ does migrate over the $CsPbBr_3$ QD active layer and then oxidize to form Ag clusters ($Ag^+ + e^- \rightarrow Ag$) at the bottom ITO cathode.

As for the migration tendency of Br, both samples exhibit fairly similar distributions from the location 2 onwards, showing a stable Br: Pb ratio of ~ 3.30. However, a significant difference between two samples is observed in the location 1. This disparity can be easily understood, as for an RRAM using Ag as the anode, the top Ag electrode has a high electrochemical activity and can preserve the Br^- ions to form $AgBr_X$ ($Ag + Br^- \rightarrow AgBr + e^-$), so that a much higher amount of Br (the Br : Pb ratio ~ 6.91) is expected in the location 1. In parallel, the bromide vacancy (V_{Br}^+) is formed inside the $CsPbBr_3$ QD active layer after Br^- anions are impelled toward the top Ag electrode under the bias. V_{Br}^+ also induces the construction of the conducting channel ($V_{Br}^+ + e^- \rightarrow V_{Br}$) and hence the subsequent transition from the HRS to the LRS in the I-V characteristics.

As a result, under the positive poling bias both the V_{Br} conducting channel and the Ag filament are well established in the RRAM and responsible for its resistive switching. Thus, the observation that much higher amounts of Ag and Br are found respectively in the location 4 and 1, is the direct evidence to prove the generation of the cations and anions, and how they are driven inside the $CsPbBr_3$ QDs by the external field. More importantly, based on the result here, the mechanism for the dynamic ionic transport and conduction processes in the perovskite LEM can be clearly outlined, as elucidated in Fig. 6b in the main text.

Supplementary Figure 6 | Influence of ITO electrode on resistive switching characteristics of $CsPbBr_3$ QD-based RRAM

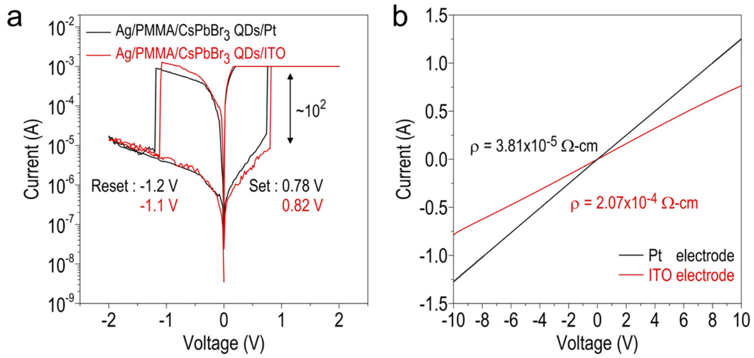

Supplementary Figure 6 a I-V characteristics of the $CsPbBr_3$ QD-based RRAM with Pt (black curve) and ITO (red curve) as the bottom electrode, respectively (compliance current to I_{CC} = 1 mA). **b** I-V measurement data for determining the electrical resistivity of the ITO (black line) and the Pt (red line) electrodes.

Supplementary Note 6

Supplementary Figure 6a shows the I-V characteristics of the $CsPbBr_3$ QD-based RRAM with Pt (black curve) and ITO (red curve) as the bottom electrode when applying a positive poling voltage (that is, Ag is the anode with Pt being the cathode). Although the Pt electrode shows a lower electrical resistivity than that of the patterned ITO electrode (see Supplementary Figure 6b), both devices exhibit almost identical I-V characteristics with the same current ratio between the HRS and the LRS of ~ 10^2. The set and reset voltages for the Ag/PMMA/$CsPbBr_3$ QD/Pt device are ~ 0.78 and ~ –1.20 V, respectively, which are similar to those of the reference device with the ITO bottom electrode (set voltage ~ 0.82 V, reset voltage ~ –1.12 V), and also comparable to the reported data of the perovskite RRAM using the Pt electrode [Adv. Electron. Mater. 1900754 (2019)]. Most importantly, the above observation confirms that the intrinsic oxygen-vacancy-rich nature inherited in the ITO electrodes barely affects the resistive switching characteristics of the $CsPbBr_3$ QD-based RRAM.

Supplementary Figure 7 | Retention and endurance tests of $CsPbBr_3$ QD-based RRAM

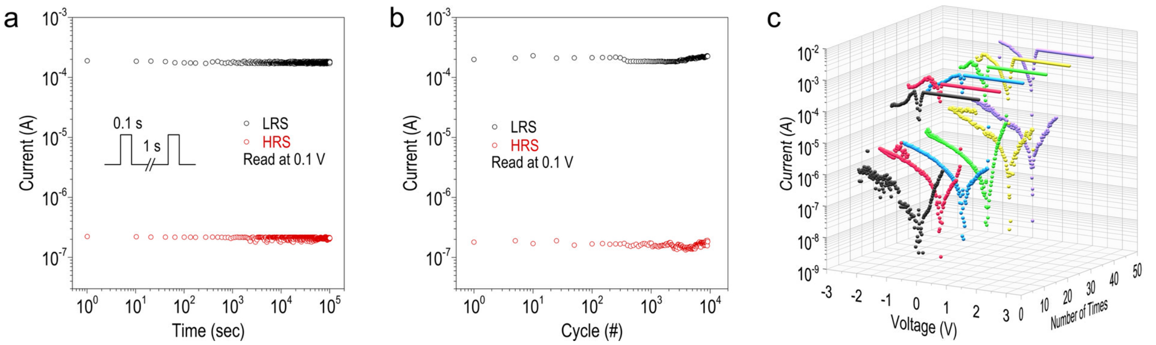

Supplementary Figure 7 a Retention performance of LRS and HRS of an Ag/$CsPbBr_3$ QDs/ITO device when acting as an RRAM. **b** Endurance test result (read at 0.1 V) of the device over 10^4 cycles. **c** I-V characteristics of the device after different numbers of times of a dc bias sweep (0 V → +2 V → 0 V → −2 V → 0 V) up to 50 times.

Supplementary Note 7

Supplementary Figure 7a shows the retention performance of the RRAM mode of a single Ag/$CsPbBr_3$ QDs/ITO device by consecutively applying square-wave bias pulses (0.1 V on a 1.1 sec period) to read the RRAM. Both the HRS and the LRS can retain for over 10^5 sec and the ON/OFF current ratio keeps at ~ 10^2, indicating good retention stability. Supplementary Figure 7b shows the endurance test results of the device under a modulated bias with a repetitive sequence of 1.5 V(Set)/ 0.1 V(Read)/ − 2.0 V(Reset) /0.1 V(Read). Except for some fluctuations in both HRS and LRS, there is no obvious decline of HRS/LRS ratio even after more than 10^4 sweeping cycles. Moreover, as shown in Supplementary Figure 7c, the RRAM can be switched reversibly between its HRS and LRS more than 50 times by repeatedly sweeping a dc bias in a sequence of 0 V → +2 V → 0 V → −2 V → 0 V. The current ratio between HRS and LRS could maintain at ~ 10^2 during these alternating positive/negative dc bias sweeps. These results altogether ensure the reliable and reproducible write/erase characteristics of either one of the Ag/$CsPbBr_3$ QDs/ITO devices in our $CsPbBr_3$ QD-based LEM when acting as an RRAM.

Supplementary Figure 8 | Device-to-device variation in set and reset voltages of $CsPbBr_3$ QD-based RRAM

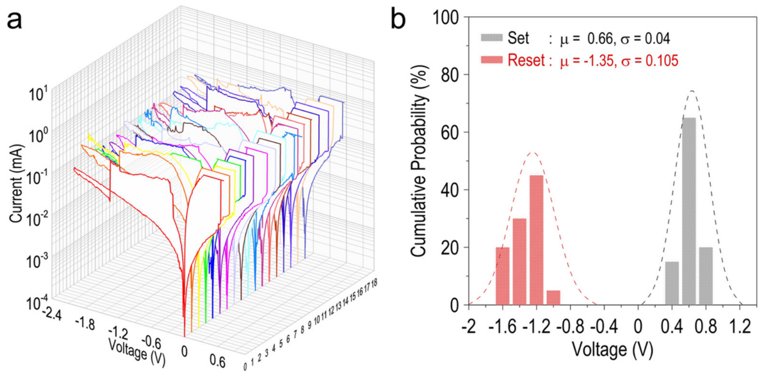

Supplementary Figure 8 a I-V characteristics of 18 $CsPbBr_3$ QD-based RRAM devices by setting the compliance current to I_{CC} = 1 mA. **b** Cumulative probability distributions of the set (right) and reset (left) voltages.

Supplementary Note 8

Supplementary Figure 8a shows the I-V characteristics of total 18 $CsPbBr_3$ QD-based RRAM devices to study the device-to-device variability in their set and reset voltages. Supplementary Figure 8b plots the cumulative probability distribution of the set and reset voltages extracted from Supplementary Figure 8a. It is clear that the set process of the RRAMs occurs mostly at the mean value of $\mu = 0.66$ V with a small standard deviation of $\sigma = 0.04$, though the reset process shows a slightly larger variability ($\sigma = 0.105$) from the mean value of $\mu = -1.35$ V. The above observations validate that our $CsPbBr_3$ QD-based RRAM exhibits a good device-to-device variability.

Supplementary Figure 9 | Electroforming process of $CsPbBr_3$ QD-based LEM

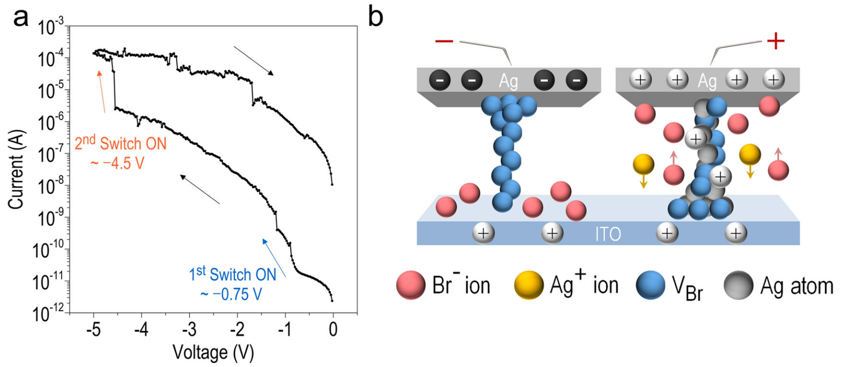

Supplementary Figure 9 a Typical I-V curve of the electroforming process of the $CsPbBr_3$ QD-based LEM. **b** Illustration of the resistive switching behavior during the electroforming process in the left and right device of the LEM.

Supplementary Note 9

To obtain the resistive switching effect, an electroforming process is needed to perform on the fabricated LEM device. Supplementary Figure 9a shows the typical I-V curve of the electroforming process of our $CsPbBr_3$ QD-based LEM. The electroforming voltage required for the LEM switching from the HRS to the LRS is ~ − 4.5 V, and a two-step hopping in the current is observed during the electroforming process (1st switch on at ~ − 0.75 V), which suggests two different types of conducting channels are induced separately in the left and right device of the LEM. Supplementary Figure 9b depicts the possible scenario of the ion migrations behind the observed resistive switching behavior in the $CsPbBr_3$ QD-based LEM during its electroforming process. After the electroforming process, both the Ag filament and V_{Br} conducting channel are well established in the right device. However, only the V_{Br} conducting channel is induced on the left since a negative bias is applied on its top Ag electrode, which hinders the migration of Ag^+ cations over the active layer to form the filament.

Supplementary Figure 10 | Energy diagram of $CsPbBr_3$ QD-based LEM under different bias conditions

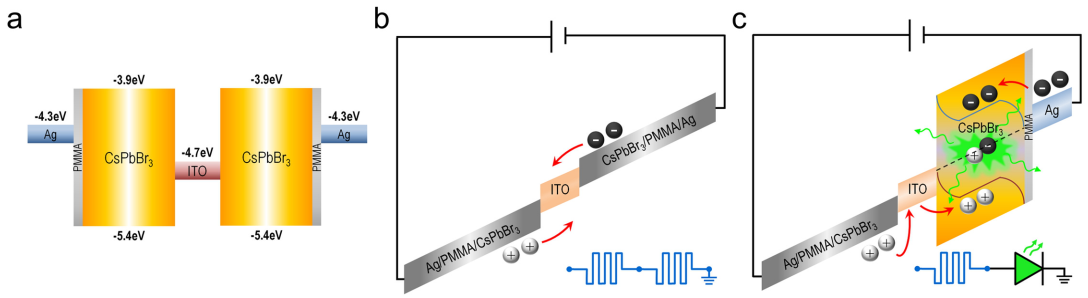

Supplementary Figure 10 Energy band diagram of the $CsPbBr_3$ QD-based LEM when the device is **a** at its initial state (i.e., no bias voltage), and when acting as **b** two series-connected ON-state RRAMs (region II), and **c** the ON-state RRAM on the left series-connected with the emitting LEC on the right (region IV).

Supplementary Note 10

Supplementary Figure 10a shows the energy band diagram of the $CsPbBr_3$ QD-based LEM at the initial state without applying positive sweep voltages. The high work function of the ITO electrode (−4.7 V) hinders the efficient electron injection in the LEM device. The movements of the ions in the active layer under an applied bias allows for the formation of Ag filament and V_{Br} conducting channel in both the right and left devices, which dramatically reduces the electrical resistance of the LEM and facilitates efficient electron injection, as shown in region II of Supplementary Figure 10b. While further increasing the applied bias to reach IV, the p-i-n homojunction is induced in the right device, whereas the left remains as an ON-state RRAM. The injected electron-hole pairs are hence recombined radiatively in the right device, resulting in visible EL emission in accordance with the bandgap energy of the $CsPbBr_3$ QDs.
